# A second species of *Cheleion* from Johor, Malaysia (Coleoptera, Scarabaeidae, Aphodiinae, Stereomerini)

**DOI:** 10.3897/zookeys.532.6116

**Published:** 2015-11-05

**Authors:** David Král, Jiří Hájek

**Affiliations:** 1 Charles University in Prague, Faculty of Science, Department of Zoology, Viničná 7, CZ-128 43 Praha 2, Czech Republic; 2 Department of Entomology, National Museum, Cirkusová 1740, CZ-193 00 Praha 9 – Horní Počernice, Czech Republic

**Keywords:** *Cheleion*, new species Coleoptera, Scarabaeidae, Aphodiinae, Stereomerini, Malaysia, Oriental Region

## Abstract

A new species of the genus *Cheleion* Vårdal & Forshage, 2010, *Cheleion jendeki* **sp. n.**, from Johor, Malaysia is described, illustrated and compared with the type species of the genus, *Cheleion malayanum* Vårdal & Forshage, 2010. Photographs of the two species are presented. The adaptation to inquilinous lifestyle of *Cheleion* is compared with those in other beetle groups and briefly discussed.

## Introduction

Scarabaeoidea (Lamellicornia) represent a distinct, cosmopolitan group of beetles, comprising approximately 2,500 genera and 35,000 species worldwide. They occupy a vast range of various niches, including inquilinous, either myrmecophilous or termitophilous, lifestyles (for a review, see Scholtz and Grebennikov 2005). Among the most peculiar presumably inquilinous scarabs are members of the small, rarely collected tribe Stereomerini of the subfamily Aphodiinae. The 21 presently known species of Stereomerini are currently assigned to nine genera. All representatives of the tribe are allegedly associated with termite nests. Seven of them, *Adebrattia* Bordat & Howden, 1995, *Australoxenella* Howden & Storey, 1992, *Bruneixenus* Howden & Storey, 1992, *Daintreeola* Howden & Storey, 2000, *Danielssonia* Bordat & Howden, 1995, *Pseudostereomera* Bordat & Howden, 1995, and *Stereomera* Arrow, 1905 are restricted to insular southeastern Asia and Australia (Howden and Storey 1992, 2000; Bordat and Howden 1995; Storey and Howden 1996; Maruyama and Nomura 2011). At present, only the genera *Cheleion* Vårdal & Forshage, 2010 and *Rhinocerotopsis* Maruyama, 2009 are known from the Peninsular Malaysia (Maruyama 2009, Vårdal and Forshage 2010). The genus *Cheleion* so far has contains only one species, *Cheleion malayanum* Vårdal & Forshage, 2010, described from Pahang. A second species, *Cheleion jendeki* sp. n., discovered from primary tropical forest of Endau Rompin NP in another Malaysian state, Johor, is described in the present paper.

## Material and methods

The specimens were examined with an Olympus SZ61 stereomicroscope. Measurements were taken with an ocular graticule. The habitus photographs were taken using a Canon MP-E 65mm f/2.8 macro lens with 5:1 optical magnification on bellows attached to a Canon EOS 550D camera. Partially focused images of specimen were combined using Helicon Focus 3.20.2Pro software. External morphology of both species was also examined with a Hitachi S-3700N environmental electron microscope in the Department of Paleontology, National Museum in Prague (in both cases using uncoated specimens). Exact label data are cited for the type material. Our remarks and addenda are found in brackets, separate label lines are indicated by a slash (/), separate labels by a double slash (//). The holotype of the newly described species is deposited in the collection of National Museum, Prague, Czech Republic (NMPC). For comparison, the holotype of *Cheleion malayanum* (deposited in Swedish Museum of Natural History, Stockholm, Sweden) was studied. For morphological terms used in the description we largely follow Howden and Storey (1992) and Vårdal and Forshage (2010).

## Taxonomy

### Cheleion jendeki sp. n.

Taxon classificationAnimaliaColeopteraScarabaeidae

http://zoobank.org/4DF33FD9-FF5D-47A1-8967-06EAEE42349D

Figures 1 , 3 , 5 , 7 , 9 , 11 , 13–15 , 17

#### Type locality.

Malaysia, Johor, Endau Rompin National Park, 02°37'12"N 103°21'00"E, 120–300 m a. s. l.

#### Type material.

Holotype: ♀, “Malaysia, Johor / Endau Rompin NP / N2.62, E103.35 / 28-31.v.2013, 120-300 m / E. Jendek & O. Šauša leg. [printed] // Cheleion jendeki sp. nov. / HOLOTYPUS ♀ / David Král & Jiří Hájek det. 2015 [red, printed]”.

#### Description of female holotype.

**Figures 1–2.**
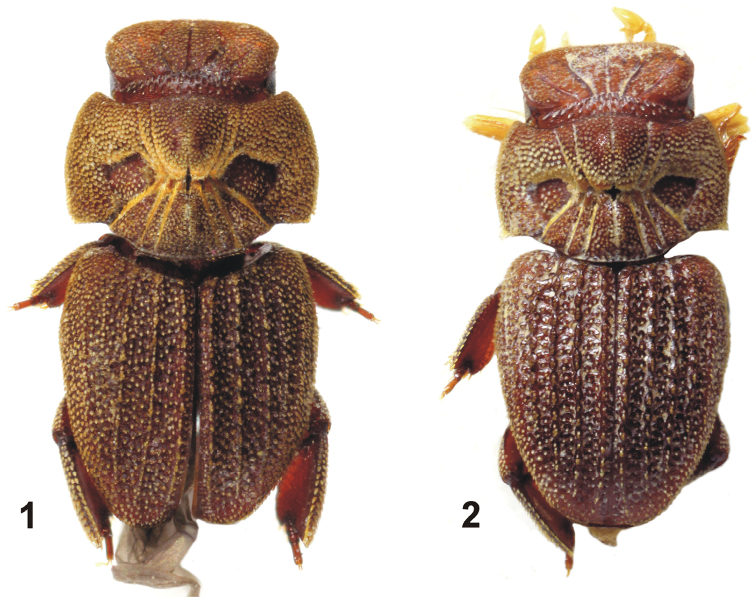
Habitus of *Cheleion*. **1** *Cheleion jendeki* sp. n. (♀ holotype) **2** *Cheleion malayanum* (♀ holotype; Malaysia, Pahang, Bukit Fraser).

Slightly convex, integument chestnut brown; head appendages and tarsi amber coloured; whole dorsal surface more or less covered with appressed lanceolate scales (Fig. 1).

**Figures 3–6.**
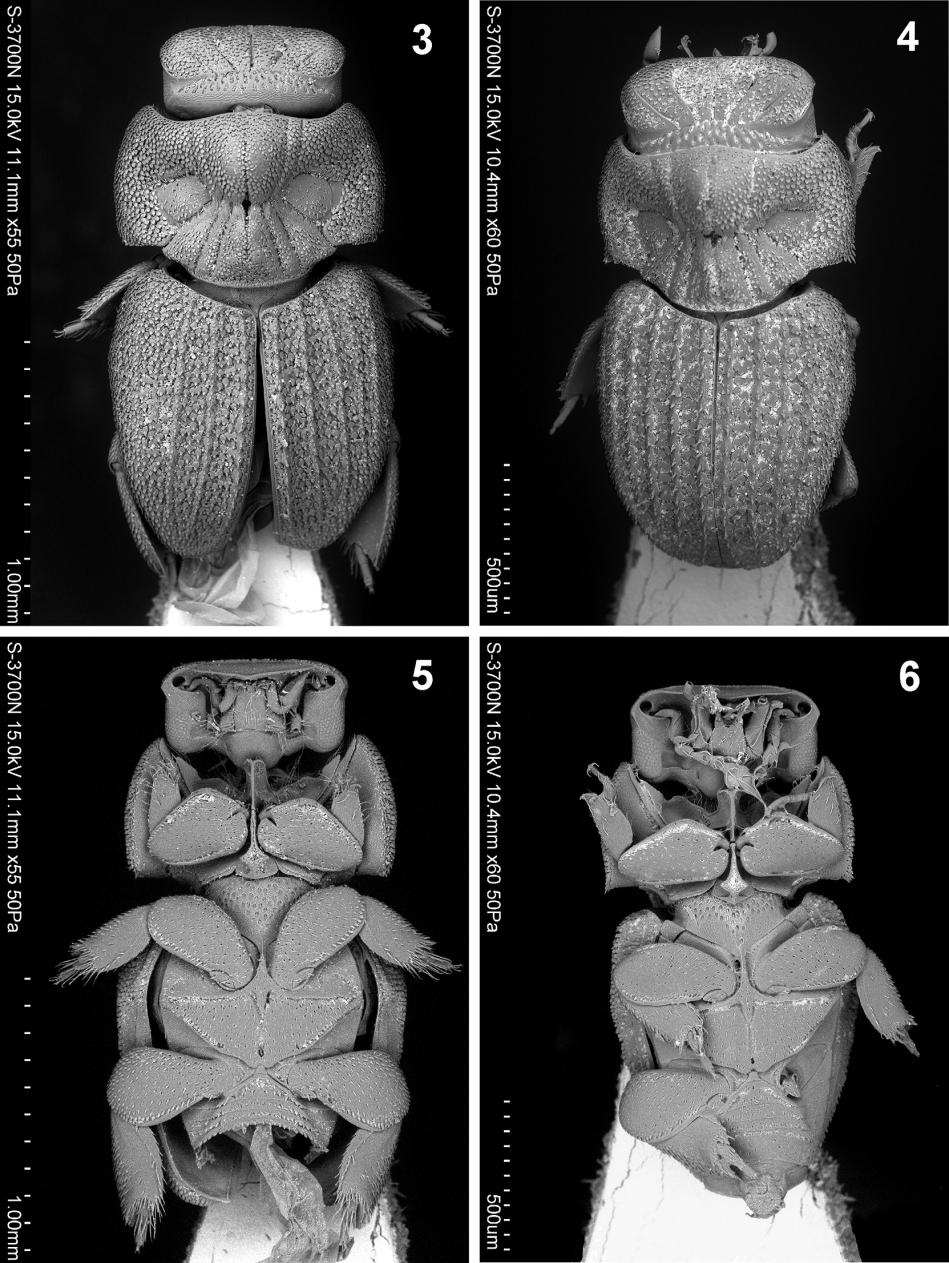
Habitus of *Cheleion*. **3, 5** *Cheleion jendeki* sp. n. (♀ holotype) **4, 6** *Cheleion malayanum* (♀ holotype; Malaysia, Pahang, Bukit Fraser) **3, 4** dorsal view **5, 6** ventral view.

**Figures 7–12.**
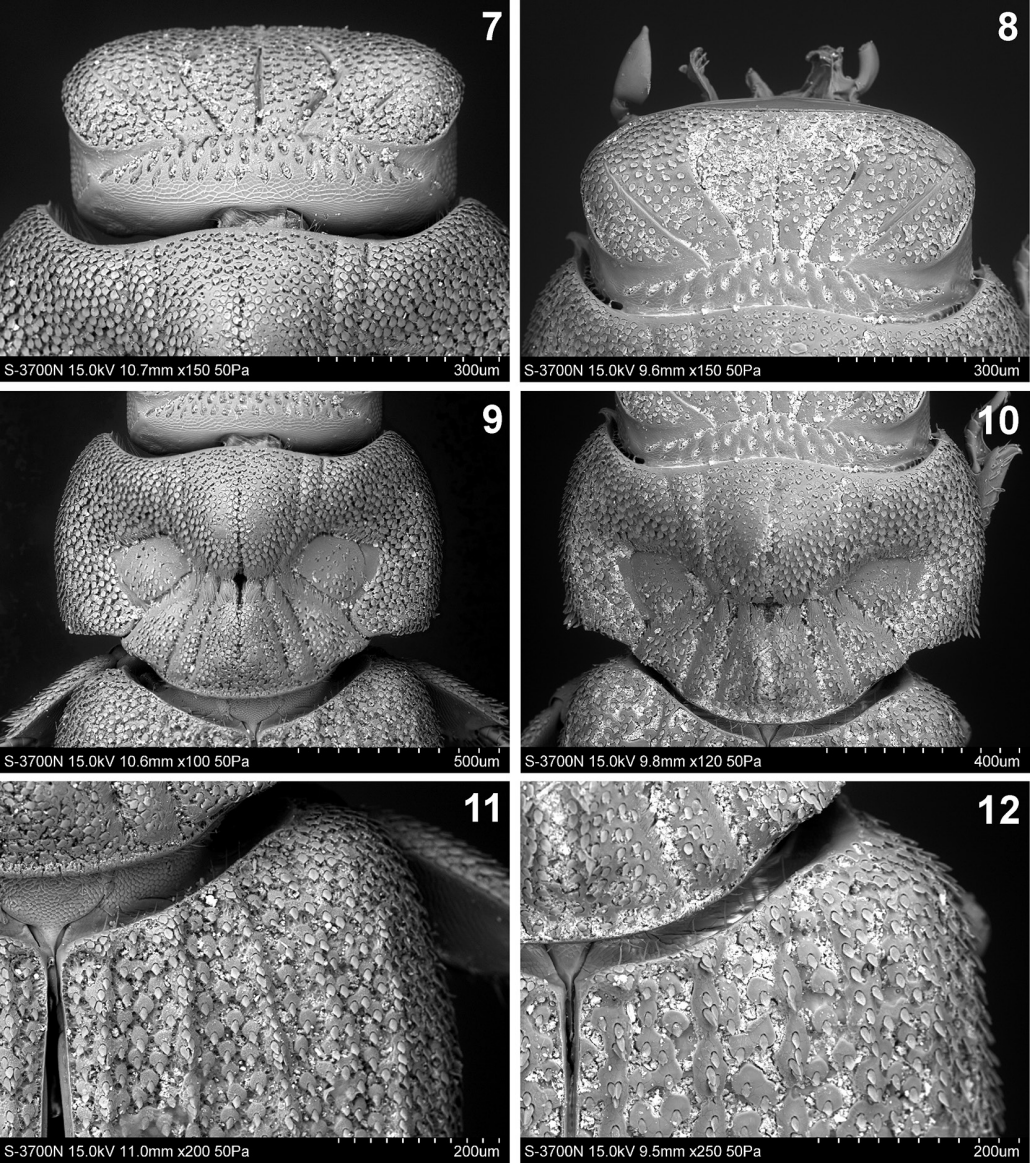
Details of *Cheleion*. **7, 9, 11** *Cheleion jendeki* sp. n. (♀ holotype) **8, 10, 12** *Cheleion malayanum* (♀ holotype; Malaysia, Pahang, Bukit Fraser) **7–8** head **9–10** pronotum **11–12** base of right elytron.

Head (Figs 1, 3, 7) remarkably transverse, subrectangular in dorsal view, clypeus shiny, impunctate, apically pointed and reflexed under head, frons slightly convex with five straight, anteriorly divergent furrows; posterior transverse furrow across head between posterolateral corners of eyes; occiput with numerous small, longitudinal pits. Surface covered with dense appressed, lanceolate, approximately regularly spaced scales, individual scales separated from each other by less than their diameter (Fig. 7). Antennae long, length equal to width of head, with long macrosetae. Maxillary palpi length equal to length of head, with securiform ultimate palpomere. Labial palpi with long macrosetae apically. Eyes small but visible in dorsal view (Fig. 7).

**Figures 13–18.**
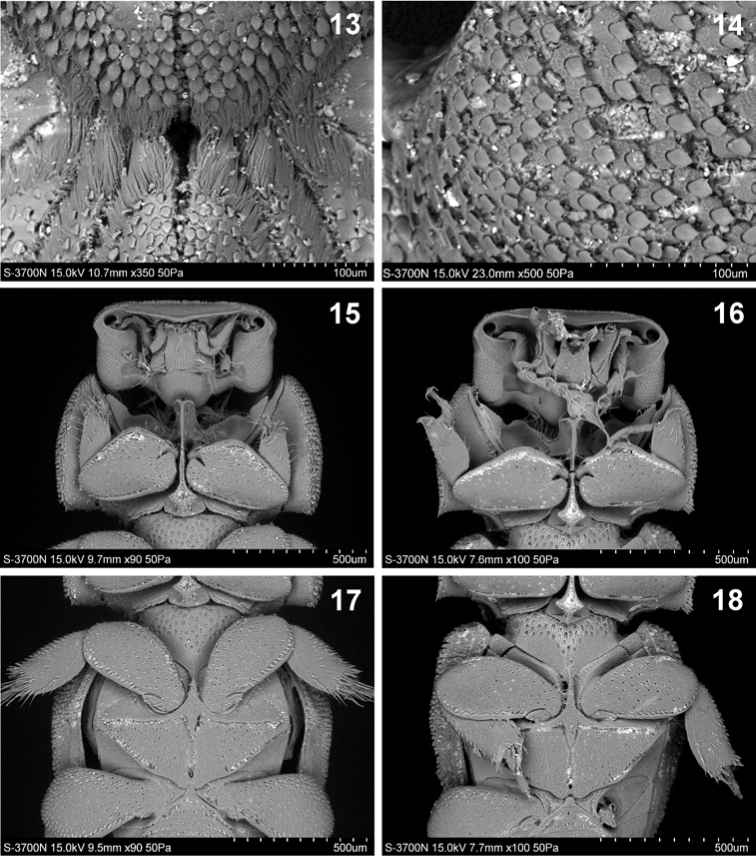
Details of *Cheleion*. **15, 17** *Cheleion malayanum* (♀ holotype; Malaysia, Pahang, Bukit Fraser) **13–14, 16, 18** *Cheleion jendeki* sp. n. (♀ holotype) **13** detail of tufts of microtrichiae (= trichomes) in centre of pronotum **14** detail of macrosetation on elytral shoulder **15–16** head and prosternum in ventral view **17–18** meso- and metaventrite in ventral view.

Pronotum (Figs 3, 9, 13) large and transverse, anterior edge shallowly bisinuate, sides regularly, broadly rounded, posterior edge with broad medial protrusion. Pronotal disc with seven furrows medially, converging towards middle in hourglass pattern, mid furrow shallower than lateral furrows (Figs 3, 9). Anteromedial disc with distinctly raised knob, posteromedial disc and posterolateral sides with slightly lower, bulbous areas; anterolaterally of the furrows with large, flat elliptical depressions, delineated by furrows. Knob posteriorly and bulbous areas anteriorly with tufts of long dense microtrichiae (= trichomes) (Fig. 13); surface covered with dense apressed, lanceolate, approximately regularly spaced scales, individual scales separated by less their diameter from each other anterolaterally and laterally; scales on knob and bulbous areas smaller and sparser; flat lateral areas with several sparse rather irregularly spaced scales only (Fig. 13).

Scutellar shield triangular, notably small (Fig. 11).

Elytra approximately as broad as pronotum and only slightly longer than pronotum and head combined; tapering posteriad, rounded apically. Each elytron with five longitudinal ridges before the lateral edge (Figs 1, 3, 11); ridges of approximately same height, elevated and almost continuous, consisting of longitudinal rows of almost confluent tubercles (Fig. 11); intervals (between ridges) flat, rugose, with irregularly circular pads, each pad bearing lanceolate scale on posterior edge, individual pads separated by less their diameter from each other discally, becoming confluent into small rows or groups laterally, especially in humeral area (Figs 11, 14). Epipleura broadly inflexed; posterior two thirds of lateral edge slightly recurved (to allow free movement of metathoracic legs).

Legs short with broad femora and tibiae; tarsi short, tetramerous; claws weak, short, almost straight (Figs 5, 15, 17). Femora shiny, covered with coarse, dense, almost regularly spaced macrosetigerous punctures (Figs 15, 17). Protibiae moderately wide, with finely serrated outer edge and one strong apical lateral tooth, tarsus inserted well before apex (Fig. 15). Meso- and metatibiae broad with finely serrated outer edge and concave apex; each with two inconspicuous terminal spurs and two rows of thick short macrosetae on outer edge (Fig. 17).

Macropterous.

Pygidium exposed, strongly punctate proximally, less strongly apically, with small emargination on proximal pygidial border.

Venter. Prosternal process remarkably elevated, strongly expanded anteriad and posteriad (Figs 5, 15); anterior part grooved longitudinally and sinuate apically, posterior part hastate, surface rugose with marginal bead (Fig. 15). Mesoventrite narrow with alutaceous surface (Fig. 17). Metaventral plate flat, triangular, tapering, widest anteriorly, grooved along midline, surface alutaceous, covered with coarse, dense, almost regularly spaced macrosetigerous punctures (Fig. 17).

Five visible abdominal ventrites apparently fused, covered with coarse, dense, almost regularly spaced macrosetigerous punctures.

#### Measurements.

Total body length: 1.9 mm, width at broadest point 0.9 mm.

#### Differential diagnosis.

The new species is classified in the genus *Cheleion* mainly by the combination of the following characters: strongly tuberculate and rugose body surface, noticeably long antennae, pronotum with distinct anteromedial knob and bulbous areas medially and laterally and hastate posterior prosternal process. *Cheleion jendeki* sp. n. is similar and probably closely related to *Cheleion malayanum*, the only other known species of the genus, but clearly differs mainly as follows:

- lateral longitudinal grooves on head straight (Figs 1, 3, 7) (weakly s-shaped in *Cheleion malayanum* (Figs 2, 4, 8));
- sides of pronotum regularly rounded, maximum width of pronotum in midlength; posterior angles subrectangular (Figs 3, 9, 15) (sides of pronotum more attenuated in basal third, maximum width of pronotum in anterior third; posterior angles obtuse with apparent spiniform scales in *Cheleion malayanum* (Figs 4, 10, 16);
- elytral surface at first sight moderately rugose (Figs 1, 3, 11) (more strongly rugose in *Cheleion malayanum* (Figs 2, 4, 12);
- elytral ridges distinctly elevated (Figs 3, 11) (almost flat in *Cheleion malayanum* (Figs 4, 12);
- elytral ridges continuous, consisting of longitudinal rows of almost confluent tubercles; lateral ridges indistinct (Fig. 3) (rather discontinuous, consisting of tubercles with scanty longitudinal groups of tubercles with scales; all elytral ridges distinct in *Cheleion malayanum* (Fig. 4));
- pads on elytral intervals separated by less their diameter and mostly arranged as triseriate discally (Fig. 11) (confluent to subconfluent and mostly arranged as biseriate in *Cheleion malayanum* (Fig. 12));
- marginal bead of posterior part of prosternal process rounded apically (Fig. 15) (angulate apically in *Cheleion malayanum* (Fig. 16)).

In spite of clear differences mentioned above, we are aware that only single specimens are known for each *Cheleion* species. In addition, both type localities are placed only about 200 km apart, without any distinct barrier between them. Thus, we cannot exclude the possibility that morphological differences of *Cheleion jendeki* sp. n. represent only an intraspecific variability of *Cheleion malayanum*, but we consider it quite improbable.

#### Etymology.

Patronymic; named in honour of our colleague and friend Eduard Jendek (Ottawa, Canada), excellent student in Buprestidae and collector of the holotype.

#### Distribution.

So far known only from the type locality in the Johor Province of continental Malaysia.

#### Collecting circumstances.

**Figure 19.**
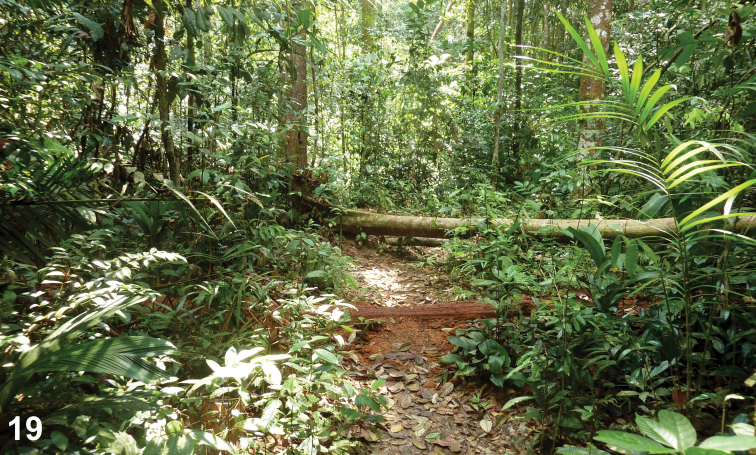
Lowland primary tropical forest in Endau Rompin NP, habitat of *Cheleion jendeki* sp. n. (photo E. Jendek).

Flight intercept trap exposed inside lowland primary tropical forest (Fig. 19; E. Jendek, pers. comm. 2015).

## Discussion

Virtually nothing is known about the biology of Stereomerini. Beetles were repeatedly supposed to be termitophilous, based on single finding of *Termitaxis holmgreni* Krikken, 1970 with termites in Peru (Krikken 1970). However this genus no longer belongs to the tribe Stereomerini as it was excluded by Bordat and Howden (1995). All other members of the Stereomerini were usually collected by flight intercept traps (FIT) in primary forests, more rarely they were also sifted or attracted at UV light (Storey and Howden 1996), collected with window trunk traps, or with yellow pan traps (Howden and Storey 2000).

We have not been able to trace any “typical characters” distinguishing myrmecophilous and termitophilous beetles. For example, Crowson (1981) noted that “termitophilous beetles tend to show rather less extreme structural modifications than comparable myrmecophilous ones”, and that “termitophilous beetles do not as a rule develop the elaborate trichomes seen in some of the more specialized myrmecophiles”. It is far beyond the scope of this paper to solve this problem, but we would like to point out several facts that may suggest myrmecophilous association of *Cheleion* and other Stereomerini.

1. There exist numerous well known myrmecophilous aphodiines, especially of the tribe Eupariini (see, e.g., Stebnicka 2009; Maruyama 2010). Those beetles usually live in debris in ant nests, fly well and are frequently collected with FIT or attracted at light.
2. In rather rare cases of presence of trichomes in termitophilous scarabaeids, those structures are not recorded from the pronotum and have a quite different appearance from *Cheleion* (see, e.g., Maruyama 2012a,b).
3. The peculiar structure on the pronotum in *Cheleion*, consisting of a central pit surrounded by numerous long microtrichia (= trichomes), is surprisingly similar to the pronotal structure of myrmecophilous ptinids (see, e.g., Bell and Philips 2008a,b), paussine carabids (Geiselhardt et al. 2007), elytral structures of myrmecophilous chlamidopsine histerids (e.g., Caterino and Degallier 2007), or elytral and abdominal structures of pselaphine (clavigerine) staphylinids (e.g., Nomura 1997, Baňař and Hlaváč 2014).

## Supplementary Material

XML Treatment for Cheleion jendeki
